# 2-(4-Chloro­phen­yl)-3-methyl-*N*-(5-methyl­thia­zol-2-yl)butanamide

**DOI:** 10.1107/S1600536808043031

**Published:** 2008-12-20

**Authors:** Jing-Li Cheng, Jin-Hao Zhao, Guo-Nian Zhu, Fu-Cheng Lin

**Affiliations:** a College of Agriculture and Biotechnology, Zhejiang University, Hangzhou 310029, People’s Republic of China; b Institute of Biotechnology, Zhejiang University, Hangzhou 310029, People’s Republic of China

## Abstract

In the title compound, C_15_H_17_ClN_2_OS, the thia­zole ring, which is essentially planar with a maximum deviation of 0.044 (3) Å, makes a dihedral angle of 54.76 (8)° with the benzene ring. In the crystal, adjacent molecules related by twofold rotation symmetry are linked by pairs of N—H⋯N hydrogen bonds.

## Related literature

For background, see: Holmstead *et al.* (1978 ▶); Forlani (1978 ▶). For a related structure, see: Zhao *et al.* (2006 ▶).
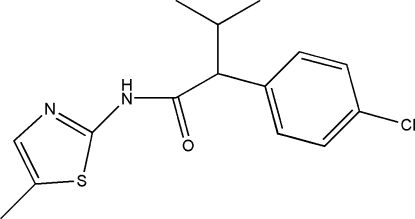


## Experimental

### 

#### Crystal data

C_15_H_17_ClN_2_OS
*M* *_r_* = 308.83
Monoclinic, 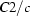

*a* = 14.9649 (6) Å
*b* = 17.6062 (7) Å
*c* = 12.5606 (5) Å
β = 99.9482 (11)°
*V* = 3259.6 (2) Å^3^
*Z* = 8
Mo *K*α radiation
μ = 0.36 mm^−1^
*T* = 298 (1) K
0.41 × 0.33 × 0.26 mm

#### Data collection

Rigaku R-AXIS RAPID diffractometer
Absorption correction: multi-scan (**ABSCOR**; Higashi, 1995 ▶) *T*_min_ = 0.858, *T*_max_ = 0.911
15655 measured reflections
3708 independent reflections
2559 reflections with *F* ^2^ > 2σ(*F* ^2^)
*R*_int_ = 0.027

#### Refinement

*R*[*F* ^2^ > 2σ(*F* ^2^)] = 0.040
*wR*(*F* ^2^) = 0.172
*S* = 1.01
3708 reflections
183 parameters
H-atom parameters constrained
Δρ_max_ = 0.27 e Å^−3^
Δρ_min_ = −0.29 e Å^−3^

### 

Data collection: *PROCESS-AUTO* (Rigaku, 1998 ▶); cell refinement: *PROCESS-AUTO*; data reduction: *CrystalStructure* (Rigaku/MSC, 2004 ▶); program(s) used to solve structure: *SIR97* (Altomare *et al.*, 1999 ▶); program(s) used to refine structure: *SHELXL97* (Sheldrick, 2008 ▶); molecular graphics: *ORTEP-3* (Farrugia, 1997 ▶); software used to prepare material for publication: *CrystalStructure*.

## Supplementary Material

Crystal structure: contains datablocks General, I. DOI: 10.1107/S1600536808043031/is2368sup1.cif

Structure factors: contains datablocks I. DOI: 10.1107/S1600536808043031/is2368Isup2.hkl

Additional supplementary materials:  crystallographic information; 3D view; checkCIF report

## Figures and Tables

**Table 1:** Hydrogen-bond geometry (Å, °)

*D*—H⋯*A*	*D*—H	H⋯*A*	*D*⋯*A*	*D*—H⋯*A*
N1—H111⋯N2^i^	0.86	2.08	2.929 (2)	168

Symmetry code: (i) 
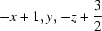
.

**Fig. 1.**
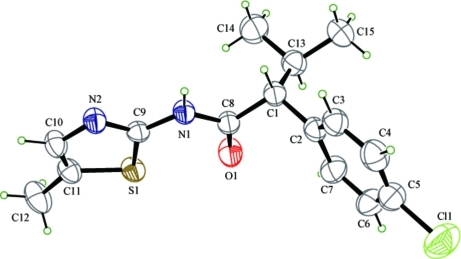
The molecular structure of (I), showing the atom-labelling scheme. Displacement ellipsoids are drawn at the 30% probability level.

**Fig. 2.**
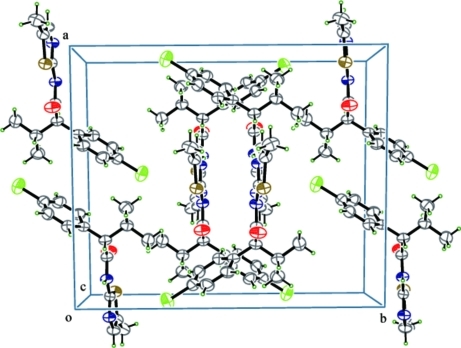
Molecular packing arrangement in the unit cell.

**Fig. 3.**
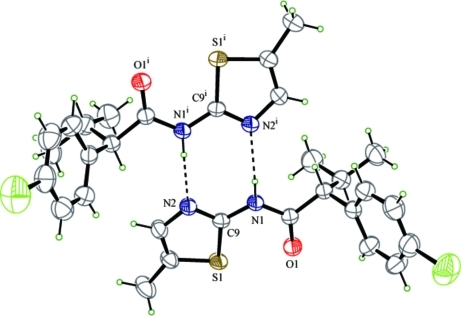
View showing the N—H···N hydrogen bonding (dashed lines) [symmetry code: (i) 1 - x, y, 3/2 - z].

## supplementary crystallographic information

### Comment

2-(4-Chlorophenyl)-3-methylbutanoyl chloride is an intermediate in the synthesis of fenvalerate, an excellent insecticide (Holmstead *et al.*, 1978). 2-Amino-5-methyl-thiazole is another heterocyclic intermediate (Forlani, 1978). As part of our continuing interest in the design and synthesis of new pesticides, we have isolated the title compound, (I), the product of the condensation reaction between 2-(4-chlorophenyl)-3-methylbutanoyl chloride and 5-methyl-2-aminothiazole, as colourless crystals suitable for X-ray analysis.

The molecular structure of (I) is illustrated in Fig. 1. Atoms N2, C10, C11, S1, C9 and N1 are coplanar, the largest deviation being 0.044 (3) Å for N1. As expected, the benzene ring is planar, and atom Cl1 lies only 0.018 (4) Å from the plane defined by the ring C atoms and itself. The angle between these two rings is 54.76 (8)°, smaller than the angle between the thiazole and benzene rings of the compound 2-(4-chlorophenyl)-3-methyl-*N*-(thiazol-2-yl) butanamide (Zhao *et al.*, 2006). There are N—H···N interactions in the crystal structure, which lead to the formation of hydrogen-bonded dimers (Figs. 2 and 3).

### Experimental

2-Amino-5-methylthiazole (1.14 g, 10 mmol), 4-dimethylaminopyridine (0.12 g), triethylamine (1.31 g) and chloroform (100 ml) were added to a 250 ml round flask. The mixture was stirred and cooled to 273 K, and then 2-(4-chlorophenyl)-3-methylbutanoyl chloride (3.47 g) was added dropwise within 30 min. The mixture was stirred at room temperature for 3 h and then 1% aqueous HCl was added (5 ml). The organic layer was washed with water to a neutral pH and dried over Na_2_SO_4_. After being filtered and concentrated, the organic residue was purified by silica-gel column chromatography, eluted with ethyl acetate-petroleum ether-formic acid (10:80:1, *v*/*v*/v), to give a white solid (yield 85%, 2.5 g), (I). It was then recrystallized from ethyl acetate-petroleum ether (2:1, *v*/*v*) to give colourless blocks (m.p. 460–461 K).

### Refinement

H atoms were included in calculated positions and refined using a riding model, with C—H distances constrained to 0.96 Å for methyl H atoms, 0.93 Å for aryl H atoms and 0.98 Å for the remainder, with N—H distances constrained to 0.86 Å, and with *U*_iso_(H) = 1.2*U*_eq_(C,N) or 1.5*U*_eq_(methyl C).

### Crystal data

**Table:**

C_15_H_17_ClN_2_OS	*F*(000) = 1296.00
*M**_r_* = 308.83	*D*_x_ = 1.258 Mg m^−^^3^
Monoclinic, *C*2/*c*	Mo *K*α radiation, λ = 0.71075 Å
Hall symbol: -C 2yc	Cell parameters from 10539 reflections
*a* = 14.9649 (6) Å	θ = 3.3–27.4°
*b* = 17.6062 (7) Å	µ = 0.36 mm^−^^1^
*c* = 12.5606 (5) Å	*T* = 298 K
β = 99.9482 (11)°	Block, colorless
*V* = 3259.6 (2) Å^3^	0.41 × 0.33 × 0.26 mm
*Z* = 8	

### Data collection

**Table:**

Rigaku R-AXIS RAPID diffractometer	2559 reflections with *F*^2^ > 2σ(*F*^2^)
Detector resolution: 10.00 pixels mm^-1^	*R*_int_ = 0.027
ω scans	θ_max_ = 27.4°
Absorption correction: multi-scan (*ABSCOR*; Higashi, 1995)	*h* = −19→19
*T*_min_ = 0.858, *T*_max_ = 0.911	*k* = −22→22
15655 measured reflections	*l* = −16→15
3708 independent reflections	

### Refinement

**Table:**

Refinement on *F*^2^	H-atom parameters constrained
*R*[*F*^2^ > 2σ(*F*^2^)] = 0.040	*w* = 1/[σ^2^(*F*_o_^2^) + (0.121*P*)^2^] where *P* = (*F*_o_^2^ + 2*F*_c_^2^)/3
*wR*(*F*^2^) = 0.172	(Δ/σ)_max_ = 0.001
*S* = 1.00	Δρ_max_ = 0.27 e Å^−^^3^
3708 reflections	Δρ_min_ = −0.29 e Å^−^^3^
183 parameters	Extinction correction: *SHELXL*
0 restraints	Extinction coefficient: 0.0028 (7)

### Special details

**Table:**

Geometry. ENTER SPECIAL DETAILS OF THE MOLECULAR GEOMETRY
Refinement. Refinement using all reflections. The weighted *R*-factor (*wR*) and goodness of fit (*S*) are based on *F*^2^. *R*-factor (gt) are based on *F*. The threshold expression of *F*^2^ > 2.0 σ(*F*^2^) is used only for calculating *R*-factor (gt).

### Fractional atomic coordinates and isotropic or equivalent isotropic displacement parameters (Å^2^)

**Table:**

	*x*	*y*	*z*	*U*_iso_*/*U*_eq_	
Cl1	0.02408 (6)	0.70387 (4)	0.59960 (9)	0.1206 (3)	
S1	0.46203 (3)	0.39223 (3)	0.44117 (3)	0.0559 (2)	
O1	0.29216 (10)	0.40983 (10)	0.48366 (10)	0.0696 (4)	
N1	0.40090 (10)	0.41256 (9)	0.63283 (11)	0.0496 (3)	
N2	0.55713 (11)	0.40575 (9)	0.63079 (12)	0.0530 (4)	
C1	0.24241 (12)	0.41401 (12)	0.65622 (13)	0.0534 (4)	
C2	0.18766 (12)	0.48677 (12)	0.64116 (13)	0.0531 (4)	
C3	0.18371 (13)	0.53424 (12)	0.72811 (17)	0.0611 (5)	
C4	0.13313 (17)	0.60024 (12)	0.7171 (2)	0.0739 (6)	
C5	0.08679 (16)	0.61988 (13)	0.6158 (2)	0.0756 (6)	
C6	0.08973 (17)	0.57396 (14)	0.5282 (2)	0.0790 (6)	
C7	0.13902 (13)	0.50774 (13)	0.54031 (16)	0.0667 (5)	
C8	0.31263 (12)	0.41250 (11)	0.58189 (13)	0.0506 (4)	
C9	0.47375 (12)	0.40501 (10)	0.57886 (13)	0.0452 (4)	
C10	0.61571 (13)	0.39512 (12)	0.55850 (16)	0.0583 (5)	
C11	0.57888 (13)	0.38625 (12)	0.45425 (16)	0.0563 (5)	
C12	0.62499 (18)	0.37260 (17)	0.35901 (19)	0.0818 (7)	
C13	0.18427 (14)	0.34156 (12)	0.63811 (17)	0.0646 (5)	
C14	0.24379 (18)	0.27066 (14)	0.6583 (2)	0.0850 (7)	
C15	0.11223 (17)	0.34038 (16)	0.7103 (2)	0.0856 (7)	
H1	0.2751	0.4131	0.7309	0.064*	
H3	0.2160	0.5213	0.7957	0.073*	
H4	0.1303	0.6309	0.7766	0.089*	
H6	0.0583	0.5877	0.4605	0.095*	
H7	0.1400	0.4765	0.4808	0.080*	
H10	0.6782	0.3942	0.5812	0.070*	
H13	0.1535	0.3409	0.5626	0.078*	
H111	0.4119	0.4175	0.7020	0.060*	
H121	0.6896	0.3732	0.3823	0.098*	
H122	0.6081	0.4118	0.3062	0.098*	
H123	0.6068	0.3241	0.3276	0.098*	
H141	0.2729	0.2694	0.7326	0.102*	
H142	0.2890	0.2719	0.6126	0.102*	
H143	0.2068	0.2262	0.6422	0.102*	
H151	0.0750	0.2960	0.6946	0.103*	
H152	0.0752	0.3850	0.6972	0.103*	
H153	0.1412	0.3393	0.7848	0.103*	

### Atomic displacement parameters (Å^2^)

**Table:**

	*U*^11^	*U*^22^	*U*^33^	*U*^12^	*U*^13^	*U*^23^
Cl1	0.1126 (6)	0.0730 (4)	0.1748 (9)	0.0221 (3)	0.0210 (5)	0.0226 (5)
S1	0.0557 (3)	0.0783 (3)	0.0340 (2)	−0.0055 (2)	0.0085 (2)	−0.00354 (19)
O1	0.0527 (8)	0.1174 (13)	0.0374 (7)	0.0055 (7)	0.0038 (5)	−0.0021 (6)
N1	0.0439 (8)	0.0725 (9)	0.0325 (7)	0.0020 (6)	0.0070 (5)	−0.0005 (6)
N2	0.0462 (8)	0.0767 (10)	0.0363 (7)	−0.0003 (6)	0.0074 (6)	−0.0030 (6)
C1	0.0451 (10)	0.0762 (12)	0.0382 (8)	0.0032 (8)	0.0052 (7)	0.0008 (8)
C2	0.0412 (9)	0.0713 (11)	0.0466 (9)	−0.0028 (8)	0.0073 (7)	0.0040 (8)
C3	0.0579 (11)	0.0705 (12)	0.0553 (10)	0.0004 (9)	0.0109 (8)	−0.0006 (9)
C4	0.0711 (15)	0.0687 (14)	0.0854 (17)	−0.0069 (10)	0.0233 (12)	−0.0088 (11)
C5	0.0608 (13)	0.0676 (13)	0.0987 (19)	−0.0022 (10)	0.0142 (12)	0.0143 (13)
C6	0.0658 (14)	0.0892 (17)	0.0771 (15)	0.0059 (11)	−0.0015 (11)	0.0222 (13)
C7	0.0589 (11)	0.0826 (14)	0.0546 (11)	0.0032 (10)	−0.0012 (8)	0.0027 (10)
C8	0.0454 (9)	0.0673 (11)	0.0388 (9)	0.0019 (7)	0.0060 (7)	0.0025 (7)
C9	0.0487 (9)	0.0531 (9)	0.0338 (7)	−0.0006 (6)	0.0071 (6)	0.0013 (6)
C10	0.0463 (10)	0.0830 (14)	0.0472 (10)	−0.0028 (8)	0.0127 (8)	−0.0056 (8)
C11	0.0564 (11)	0.0683 (11)	0.0465 (10)	−0.0072 (8)	0.0159 (8)	−0.0059 (8)
C12	0.0754 (15)	0.118 (2)	0.0586 (12)	−0.0162 (14)	0.0299 (11)	−0.0212 (13)
C13	0.0569 (11)	0.0775 (13)	0.0589 (11)	−0.0026 (9)	0.0083 (9)	0.0043 (10)
C14	0.0848 (17)	0.0730 (15)	0.0990 (19)	0.0032 (12)	0.0214 (14)	0.0015 (13)
C15	0.0702 (15)	0.0908 (17)	0.1022 (19)	−0.0038 (12)	0.0331 (13)	0.0192 (14)

### Geometric parameters (Å, °)

**Table:**

Cl1—C5	1.744 (2)	C13—C14	1.529 (3)
S1—C9	1.7227 (17)	C13—C15	1.524 (3)
S1—C11	1.731 (2)	N1—H111	0.860
O1—C8	1.220 (2)	C1—H1	0.980
N1—C8	1.365 (2)	C3—H3	0.930
N1—C9	1.386 (2)	C4—H4	0.930
N2—C9	1.304 (2)	C6—H6	0.930
N2—C10	1.380 (2)	C7—H7	0.930
C1—C2	1.515 (2)	C10—H10	0.930
C1—C8	1.522 (2)	C12—H121	0.960
C1—C13	1.539 (2)	C12—H122	0.960
C2—C3	1.385 (2)	C12—H123	0.960
C2—C7	1.397 (2)	C13—H13	0.980
C3—C4	1.381 (3)	C14—H141	0.960
C4—C5	1.384 (3)	C14—H142	0.960
C5—C6	1.372 (3)	C14—H143	0.960
C6—C7	1.374 (3)	C15—H151	0.960
C10—C11	1.339 (2)	C15—H152	0.960
C11—C12	1.500 (3)	C15—H153	0.960
			
C9—S1—C11	89.24 (9)	C13—C1—H1	107.7
C8—N1—C9	123.37 (14)	C2—C3—H3	119.1
C9—N2—C10	109.35 (15)	C4—C3—H3	119.1
C2—C1—C8	110.74 (16)	C3—C4—H4	120.6
C2—C1—C13	113.75 (15)	C5—C4—H4	120.6
C8—C1—C13	109.12 (16)	C5—C6—H6	119.9
C1—C2—C3	120.52 (15)	C7—C6—H6	119.9
C1—C2—C7	121.50 (17)	C2—C7—H7	119.7
C3—C2—C7	117.97 (19)	C6—C7—H7	119.7
C2—C3—C4	121.75 (19)	N2—C10—H10	121.4
C3—C4—C5	118.8 (2)	C11—C10—H10	121.4
Cl1—C5—C4	119.5 (2)	C11—C12—H121	109.5
Cl1—C5—C6	119.8 (2)	C11—C12—H122	109.5
C4—C5—C6	120.6 (2)	C11—C12—H123	109.5
C5—C6—C7	120.1 (2)	H121—C12—H122	109.5
C2—C7—C6	120.7 (2)	H121—C12—H123	109.5
O1—C8—N1	121.86 (17)	H122—C12—H123	109.5
O1—C8—C1	122.81 (15)	C1—C13—H13	108.2
N1—C8—C1	115.30 (14)	C14—C13—H13	108.2
S1—C9—N1	123.45 (12)	C15—C13—H13	108.2
S1—C9—N2	115.22 (14)	C13—C14—H141	109.5
N1—C9—N2	121.32 (15)	C13—C14—H142	109.5
N2—C10—C11	117.29 (18)	C13—C14—H143	109.5
S1—C11—C10	108.91 (16)	H141—C14—H142	109.5
S1—C11—C12	122.03 (14)	H141—C14—H143	109.5
C10—C11—C12	129.06 (19)	H142—C14—H143	109.5
C1—C13—C14	110.73 (18)	C13—C15—H151	109.5
C1—C13—C15	111.30 (18)	C13—C15—H152	109.5
C14—C13—C15	110.0 (2)	C13—C15—H153	109.5
C8—N1—H111	118.3	H151—C15—H152	109.5
C9—N1—H111	118.3	H151—C15—H153	109.5
C2—C1—H1	107.7	H152—C15—H153	109.5
C8—C1—H1	107.7		
			
C9—S1—C11—C10	−0.73 (16)	C13—C1—C2—C7	−65.9 (2)
C9—S1—C11—C12	178.9 (2)	C8—C1—C13—C14	57.6 (2)
C11—S1—C9—N1	−178.04 (16)	C8—C1—C13—C15	−179.67 (16)
C11—S1—C9—N2	0.70 (15)	C13—C1—C8—O1	60.1 (2)
C8—N1—C9—S1	−1.7 (2)	C13—C1—C8—N1	−118.44 (17)
C8—N1—C9—N2	179.66 (17)	C1—C2—C3—C4	−178.9 (2)
C9—N1—C8—O1	−4.1 (2)	C1—C2—C7—C6	−180.0 (2)
C9—N1—C8—C1	174.50 (16)	C3—C2—C7—C6	0.7 (3)
C9—N2—C10—C11	−0.2 (2)	C7—C2—C3—C4	0.4 (3)
C10—N2—C9—S1	−0.4 (2)	C2—C3—C4—C5	−1.3 (3)
C10—N2—C9—N1	178.33 (16)	C3—C4—C5—Cl1	−178.89 (19)
C2—C1—C8—O1	−65.9 (2)	C3—C4—C5—C6	1.1 (3)
C2—C1—C8—N1	115.61 (17)	Cl1—C5—C6—C7	179.97 (13)
C8—C1—C2—C3	−123.34 (19)	C4—C5—C6—C7	−0.0 (3)
C8—C1—C2—C7	57.4 (2)	C5—C6—C7—C2	−0.9 (3)
C2—C1—C13—C14	−178.17 (17)	N2—C10—C11—S1	0.7 (2)
C2—C1—C13—C15	−55.5 (2)	N2—C10—C11—C12	−178.9 (2)
C13—C1—C2—C3	113.3 (2)		

### Hydrogen-bond geometry (Å, °)

**Table:**

*D*—H···*A*	*D*—H	H···*A*	*D*···*A*	*D*—H···*A*
N1—H111···N2^i^	0.86	2.08	2.929 (2)	168

Symmetry codes: (i) −*x*+1, *y*, −*z*+3/2.
